# Sperm Cryopreservation in Canaries to Protect Endangered Songbird Species: Comparison of Different Cryoprotectants

**DOI:** 10.1002/vms3.70101

**Published:** 2024-10-30

**Authors:** Arda Onur Özkök, Burcu Esin, Eser Akal

**Affiliations:** ^1^ Department of Veterinary Amasya University Suluova Vocational School Amasya Turkey; ^2^ Department of Reproduction and Artificial Insemination Faculty of Veterinary Medicine University of Ondokuz Mayis Samsun Turkey

**Keywords:** cryoprotectant, Gloster canary, sperm cryopreservation

## Abstract

Sperm cryopreservation is a rather complex process that needs to be adapted to wild and domestic bird species to ensure adequate efficiency. This study aimed to determine the usability of different cryoprotectants in the cryopreservation of Gloster canary sperm. For this purpose, sperm samples were collected from 12 2‐year‐old male Gloster canaries three times a week using cloacal massage for 4 weeks. After individual evaluation, sperm samples from the canaries were combined. Mixed sperm were divided into two groups in the study. Overall, 8% dimethyl sulfoxide (DMSO) and ethylene glycol (EG) were used as cryoprotectants. Sperm samples were drawn into straws after adding Dulbecco's Modified Eagle Medium (DMEM) extender with high glucose ratio and two different cryoprotectants in a 1:1 ratio and frozen to −80°C with liquid nitrogen vapour and then stored in liquid nitrogen at −196°C. Frozen‐thawed semen samples were evaluated regarding motility, vitality, plasma membrane integrity (hypoosmotic swelling test [HOST]), density and abnormal spermatozoa rate. The highest motility value after freezing and thawing was determined in the EG group with 31.667% ± 4.773%. In addition, vitality, plasma membrane integrity and normal sperm morphology were statistically significantly higher in the EG‐frozen group, whereas head and tail abnormality was low (*p* < 0.05). This study determined that a DMEM extender containing 8% EG was more advantageous than a DMEM containing DMSO regarding spermatological parameters and could be used for long‐term storage of canary sperm.

## Graphical abstract

Sperm collected from two study groups were frozen with diluent containing DMSO and EG after dilution. At the end of storage, thawed semen samples were evaluated for spermatological parameters, and EG was more successful than DMSO in storing songbird sperm.

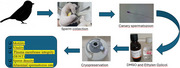

## 1 Introduction

Many finch species are seriously endangered in various parts of the world. The Gouldian finch (*Erythrura gouldiae*), a bird endemic to Australia, is at risk of extinction (Day et al. 2019). The Australian tree finch (*Camarhynchus pauper*) was included in the red list category by the International Union for Conservation of Nature in 2009 (O'Connor et al. 2010). The Mangrove finch (*Camarhynchus heliobates*) and the Black‐throated finch (*Poephila cincta cincta*) are other examples of critically endangered species (Vanderduys et al. 2016). Artificial insemination is used for agricultural production or to protect some endangered wild birds. These applications generally include the short‐term and long‐term storage of sperm by diluting it with fresh sperm (Samour 2004). Significant reproduction‐related problems such as physical or behavioural disorders, problems related to sexual incompatibility and preservation of genetic diversity in birds can be solved by artificial insemination (Blanco et al. 2009).

Sperm samples collected for artificial insemination can be stored by freezing. In this way, it is possible to protect genetic resources (Gee et al. 2004). Developing reproductive technologies such as artificial insemination and cryopreservation methods in birds is necessary to protect species at risk of extinction and long‐term genetic studies (Carreira et al. 2022). Long‐term freezing of sperm is significant for the protection of species at risk of extinction (Prieto et al. 2014). However, despite many studies on the commercial use of sperm in poultry and the preservation of genetic resources, the cryopreservation of sperm in poultry is still not at the desired level as in mammals (Asano and Tajima 2017). The cell membrane of poultry sperm contains more unsaturated fatty acids than mammals. Due to this situation, they are more vulnerable to oxidative stress elements during the freeze‐thaw protocol compared to mammals. Therefore, more antioxidants are needed. In addition, poultry sperm is more sensitive to the toxic effects of cryoprotectants used during freezing. It is essential to use an effective cryoprotectant agent in poultry (Çiftci and Aygün 2018).

Studies on the storage and use of semen in poultry animals are limited. However, studies on the cryopreservation of semen have gained importance in protecting economically valuable species and revealing desired genetic characteristics (Blesbois and Brillard 2007). The cryopreservation of bird sperm allows the storage of genetic resources cost‐effectively. However, it is known that the cryopreservation of bird sperm can vary among different breeds and lines where genetic studies are conducted (Woelders, 2021). In addition, as the sensitivity of the sperm of different bird species to the cryopreservation process is different, it is not possible to achieve the same success rate in the cryopreservation of semen for each bird species (Blesbois and Brillard 2007).

Cryoprotectant agents are chemical substances that protect cells and tissues against damage caused by intracellular ice crystal formation. They show their effects by affecting the cell membrane. Although less toxic, their protective effects are limited (Stewart, Langer, and Jensen 2018). The main reason the success rate in cryopreservation of sperm in poultry has not been achieved as in mammals is that the cell membrane of poultry sperm is more fluid than in mammals. In addition, the cell membrane of poultry sperm contains more polyunsaturated fatty acids. Therefore, using the appropriate cryopreservant agent is crucial (Janosikova et al. 2023). Dimethyl sulfoxide (DMSO) has been successfully used in the cryopreservation of many different species of bird sperm (Rakha et al. 2018). It has been observed that DMSO has different effects when used in different proportions. When used at low concentrations, it causes the cell membrane structure to thin, increasing intracellular fluidity, whereas higher doses cause water droplets to form on the cell membrane. As the DMSO rate increases, lipid molecules in the cell membrane dissociate, causing degeneration in the cell (Gurtovenko and Anwar 2007). Therefore, with the increase in the DMSO rate, the entry of water and calcium (Ca) ions into the cell in the plasma increases, making it possible to increase cell permeability (de Ménorval et al. 2012). For this reason, changing the DMSO concentration can indirectly change the effect of the cryoprotectant (Gurtovenko and Anwar 2007). Ethylene glycol (EG) cannot stop the cell membrane passages that effectively form ice crystals. However, it reduces the harmful effect by ensuring that this effect occurs gradually in the broader area (Sieme, Oldenhof, and Wolkers 2015). As EG has a minimal molecular structure, it can pass through the plasma membrane faster. For this reason, it is thought that EG may have a more protective effect than many other cryoprotectant agents (Taşdemir et al. 2013).

This study aimed to investigate the effects of EG and DMSO cryoprotectants on freezing and thawing of Gloster canary spermatozoa during cryopreservation.

## 2 Materials and Methods

### 2.1 Experimental Plan and Management

Canary is a songbird in the class (order Passeriformes, suborder Oscines). Like other finch species, it has 2*n* = 80 chromosomes (Santos et al. 2017). For this reason, 12 active male Gloster canaries aged 2 years and weighing an average of 28 g were used in the study. The canaries were housed in 4‐story cages measuring 60 cm × 40 cm × 50 cm. The canaries used in the study were fed plain canary food ad libitum throughout the study, and no additional food supplements were applied. To induce sexual activation, a photoperiod of 16 h light and 8 h dark (Singh, Montoure, and Ketterson 2021) was applied. Necessary care was taken regarding the care and welfare of the animals used in the study. All kinds of stressful environments were avoided while collecting sperm from male birds.

### 2.2 Sperm Collection and Preparation

After the photoperiod application, canaries were trained by collecting semen every 4 days for 3 weeks to eliminate the adverse effects that may occur due to stress during the semen collection process. Sperm collection was performed using the cloacal massage method (Cramer et al. 2020). Before sperm cryopreservation, motility, vitality, plasma membrane integrity (hypoosmotic swelling test [HOST]), density and abnormal spermatozoa rate were evaluated and noted in fresh semen for each bird. It was observed that motility was over 70%. The vitality rate and plasma membrane integrity were determined to be over 90%. It was observed that the average sperm density was 85 million, and the abnormal spermatozoa rate was below 10%. After individual evaluation, sperm samples taken from canaries were combined.

### 2.3 Sperm Cryopreservation Protocol

As canary sperm is quickly affected by cold shock due to external environmental differences, as well as the stressful environment that may occur during dilution and freezing, sperm samples taken for each bird were immediately transferred to a 35°C hot water bath and diluted with an 8% cryoprotectant‐containing diluent. Dulbecco's modified Eagle's medium (DMEM) diluents with high glucose concentration were used as diluents in the study. The DMEM diluent used contained 4500 mg/L glucose, 110 mg/L sodium pyruvate and l‐glutamine (Mora et al. 2017). The mixed semen samples were diluted 1:1 in high glucose DMEM extender in the presence of 8% EG or 8% DMSO, drawn into 0.25 mL straws and kept in the refrigerator for equilibration at +5°C for 15 min. As the sperm samples in the straws were in low quantities, they were drawn into straws according to the procedure applied to honeybees in the form of extender‐air gap‐sperm‐air gap‐extender to prevent any adverse effects that may occur during strawing (Hopkins, Herr, and Sheppard 2012). After the equilibration process, the sperm straws were kept in liquid nitrogen vapour at −130°C for 10 min, and the temperature was reduced, then transferred to liquid nitrogen at −196°C (Iaffaldano, Di Iorio, and Rosato 2012). Frozen semen samples were thawed after being stored for 1 month. The samples were thawed at 37°C for 30 s and evaluated (Murugesan and Mahapatra 2020).

### 2.4 Assessment of Sperm Variables

Spermatological parameters were evaluated under the phase‐contrast microscope (Nikon, Eclipse E200, Tokyo, Japan) equipped with a heating stage at 37°C.

#### 2.4.1 Assessment of Motility

After the diluted 10 mL sperm samples were thawed in a hot water bath at 37°, total motility was evaluated by examining them for 1 min under a pre‐heated microscope (Nikon, Eclipse, Tokyo, Japan) at 400× magnification with the help of a computer‐aided sperm analyser (CASA) system (Yang et al. 2019).

#### 2.4.2 Assessment of Viability

Each sperm sample was thawed in a hot water bath at 37°C and gently mixed with eosin nigrosin dye. A smear was taken and dried immediately. At least 200 spermatozoons were counted at 400× magnification. Those who received partial or complete dye were considered dead. Those who did not receive dye were considered alive (Bakst and Cecil 1997).

#### 2.4.3 Evaluation of Plasma Membrane Integrity

A HOST for plasma membrane integrity was used to evaluate. For this purpose, the semen sample was diluted with hypoosmotic solution (100 mOsm/L, 0.735 g sodium citrate + 1 g fructose + 100 mL distilled water) at a ratio of 1:10 and incubated at 37°C for 60 min and then examined under a pre‐heated light microscope at 37°C for 400 min. At least, 100 sperm cells were counted and examined at magnification. Sperm cells with swollen and curved tails were considered HOST‐positive (Zubair, Ahmad, and Jamil 2015).

#### 2.4.4 Evaluation of Spermatozoa Morphology

A volume of 2 µL semen sample from each canary was transferred in 20 µL phosphate buffer saline (PBS) solution containing 5% formaldehyde. Then, 10 µL of sperm sample, spread on the slide, was left to dry at a 45° angle. After drying, the slide was dipped in the jar containing SpermBlue dye and left for 2 min. After the slide was painted with SpermBlue, it was left to dry at 60°–80°. After drying, the slide dipped twice in a jar containing distilled water and was left to dry again (Cramer et al. 2020). After the staining process, Gloster canary sperm morphology was analysed using a CASA at 40× magnification (Nikon, Eclipse, Tokyo, Japan) for at least ten spermatozoa (Fıgure 1).

**FIGURE 1:**
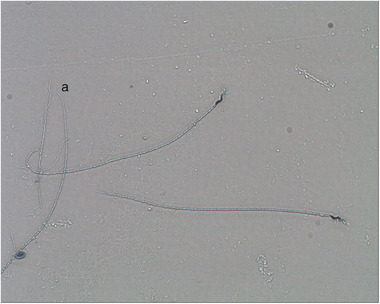
Abnormal spermatozoon [(a) indicates head anormality].

### 2.5 Statistical Analysis

Statistical analysis of data (motility, viability, plasma membrane functional integrity and spermatozoa morphology) obtained in this study was evaluated using independent samples *t*‐test with SPSS 21.0 (IBM Corp. 2011) package programme. The effects of the groups were considered significant at the *p* < 0.05.

## 3 Results

In the study, EG and DMSO cryoprotectant agents were added to the sperm extender taken for both groups and frozen. After being stored for 1 month, they were thawed and evaluated regarding relevant spermatological parameters. The effects of cryoprotectant agents on spermatological parameters are presented in Table 1. The study showed no statistically significant difference in motility and sperm density values. Plasma membrane integrity is an important parameter indicating that sperm is healthy. It is determined by the HOST. As the HOST positive rate increases, the reliability of sperm increases. The increase in the HOST positive rate in the EG group was statistically very significant. In the frozen and thawed canary sperm, the significant increase in the vitality rate in the EG group was remarkable (*p* ≤ 0.001). In the study, the vitality rates were examined immediately after equilibration, freezing and thawing. Although no significant difference was observed after equilibration, a significant difference was observed regarding vitality rate in the frozen and thawed sperm. In addition, the morphological examination determined a significant increase in abnormal spermatozoa rate in the DMSO group. In the evaluation made between the spermatozoon parts in determining the rate of abnormal spermatozoon in the EG and DMSO groups, it was seen that the head and flagellum parts of the DMSO group were significantly more negatively affected (Table 2). The increase in abnormalities due to flagellum damage, in particular, is surprising. As a result, it is thought that EG added to the sperm extender at a rate of 8% is advantageous compared to DMSO added at a rate of 8% in the cryopreservation of canary sperm.

**TABLE 1:** Effect of cryoprotectants on spermatological parameters (*N* = 6).

Groups	Motility	Plasma membrane integrity	Viability	Abnormal sperm	Sperm count	Viability (equilibration)
EG	31.667 ± 4.773	88.00 ± 0.730	42.17 ± 1.579	29.333 ± 3.720	82.00 ± 2.033	85.00 ± 2.017
DMSO	26.667 ± 4.944	70.33 ± 1.282	35.33 ± 0.919	42.167 ± 2.994	82.33 ± 1.022	77.83 ± 2.926
*p*	0.484	≤0.001	<0.05	≤0.001	0.726	0.071

*Note*: Data are expressed as means ± SD; significance: *p*.

Abbreviations: DMSO, dimethyl sulfoxide; EG, ethylene glycol.

**TABLE 2:** Evaluation of morphological parts of sperm (*N* = 6).

Groups	Acrosome	Nucleus	Head	Flagellum
EG	16.833 ± 1.833	3.833 ± 0.401	3.167 ± 0.401	5.500 ± 1.544
DMSO	21.833 ± 1.642	4.667 ± 0.333	5.667 ± 0.615	10.167 ± 1.667
*p*	0.070	0.141	<0.05	<0.05

*Note*: Data are expressed as means ± SD; significance: *p*.

Abbreviations: DMSO, dimethyl sulfoxide; EG, ethylene glycol.

In the study, it was seen that there was a positive correlation between some parts of sperm cells morphologically (Table 3). It was seen that acrosome significantly correlated with the nucleus, head and flagellum. Remarkably, there was a significant positive correlation between flagellum and head and acrosome. Therefore, the positive correlation between the canary spermatozoon parts suggests that the negativities seen in these parts may directly affect other parts of the spermatozoon. This shows that canary spermatozoon may be more affected by the negativities related to freezing‐thawing and environmental differences. It reveals the importance of cryoprotectant agents for these negativities.

**TABLE 3:** Correlations between parts of spermatozoon (*N* = 12).

Trait	Acrosome length (µm)	Nucleus length (µm)	Head length (µm)	Flagellum length (µm)
Acrosome length (µm)	…	0.604^*^ (0.037)	0.669^*^ (0.017)	0.832^**^ (0.001)
Nucleus length (µm)	0.604^*^ (0.037)	…	0.410 (0.186)	0.551 (0.064)
Head length (µm)	0.669^*^ (0.017)	0.410 (0.186)	…	0.722^**^ (0.008)
Flagellum length (µm)	0.832^**^ (0.001)	0.551 (0.064)	0.722^**^ (0.008)	…

^*^ The correlation is important.

^**^ The correlation is very important.

## 4 Discussion

In this study, the effects of two different cryoprotectant agents on spermatological parameters during cryopreservation of songbird sperm were investigated. For this purpose, the effects of 8% DMSO and EG added to the sperm extender on canary spermatozoa were evaluated. EG was more successful in motility and viability for canary spermatozoa. DMSO and EG penetrate the cell and prevent the formation of intracellular ice crystals. Therefore, they minimize the damage that intracellular ice crystals will cause during freezing (Benesova and Trefil 2016). Although used in different doses, it was reported that 8% DMSO was successful in the viability of poultry sperm during freezing and thawing compared to glycerol. In addition, it was observed that the fertilization rate of sperm diluted with an 8% DMSO‐added extender increased compared to glycerol (Rakha et al. 2018). Therefore, 8% DMSO was used for cryoprotectant addition. Another cell‐penetrating cryoprotectant substance to be compared with DMSO in the study was 8% EG. In a cryopreservation study conducted on a budgerigar species, it was observed that EG provided better results in post‐thaw spermatological parameters compared with Dimethylacetamide (DMA). Total motility was 24.1 ± 8.6 in the DMA‐frozen group and 27.4 ± 10.4 in the EG‐frozen group (Gloria et al. 2014). Therefore, the effects of 8% DMSO and EG were compared in our study.

In a study examining the cryoprotective effects of different cryoprotectant agents on chicken sperm, 8% DMSO and 8% EG were used. No significant difference was observed in total motility (*p* > 0.05). In addition, the flow cytometry analysis method examined spermatozoa damage (apoptotic spermatozoa rate), and no significant difference was found between the DMSO and EG groups (Svoradová et al. 2018). In our study, abnormal spermatozoa rate and motility were evaluated. Although no significant difference was observed in motility values, a significant increase was observed in the abnormal spermatozoa rate in the DMSO group. Although the abnormal spermatozoa rate increased significantly in our study, the lack of a statistically significant difference between the motility values is thought to be due to the very low total motility in both groups. It is also known that sperm sensitivity may vary in different bird species (Blesbois and Brillard 2007). Therefore, canary sperm is likely to be more sensitive than chicken sperm. In another study, due to the adverse effects of glycerol used for cryopreservation on fertilization in Indian red jungle fowl (*Gallus gallus murghi*) sperm, the effects of different doses of DMSO on sperm motility, plasma membrane integrity, sperm viability rate and allosome integrity parameters were investigated as an alternative (Rakha et al. 2018).

Among the 0%, 4%, 6%, 8% and 10% DMSO alternatives added to the extender, the most reliable result was obtained from 8% DMSO. Notably, the study's values were 60% and above for motility, plasma membrane integrity and sperm vitality. In our study, 8% DMSO was compared with 8% EG regarding sperm motility, plasma membrane integrity and vitality rate. However, it was observed that spermatological parameters were significantly lower after freezing and thawing for both groups. In addition to the fact that chicken sperm is more durable than canary sperm, the compatibility of the extender used in the study with the sperm is also essential. In the study, the time the frozen sperm was stored per straw was not mentioned in detail. Therefore, it can be concluded that the breed, the extender used, storage conditions and duration may be effective in the frozen storage of sperm. In a study conducted with BEC chickens, the effects of EG, dimethyl formamide and dimethyl acetamide cryoprotectants added to the extender were compared. It was reported that the best viability rate after thawing was obtained in spermatozoa diluted with EG in a cryopreservation study conducted with a slow freezing protocol (Váradi et al. 2013). However, approximately half of the viable spermatozoa consisted of morphologically abnormal spermatozoa.

Similarly, our study observed a significant increase in the abnormal spermatozoa rate after freezing and thawing. This suggests that poultry spermatozoa may be more quickly affected by adverse conditions such as oxidative stress, pH of the environment and temperature changes encountered during freezing and thawing than mammalian spermatozoa. In a study on the cryopreservation of chicken sperm, the effects of 6% dimethylformamide (DMF), 2% DMSO and 8% EG on fertilization were investigated. It was observed that the highest rate of fertilization was achieved in the EG group among the different cryoprotectants added to the diluted semen (Murugesan and Mahapatra 2020). In another study, the effects of 6% DMF, 9% DMA and 8% EG cryoprotectants added to the Beltsville Poultry Semen Extender (BPSE) extender on motility, vitality, abnormal spermatozoa, sperm acrosomes and fertility in chicken sperm were evaluated. Similar to other studies, a decrease in spermatological parameters and fertilization rates was observed in frozen and thawed sperm. In addition, the combined use of cryoprotectant agents had a negative effect. The study reported that 8% EG was more advantageous than other cryoprotectants and different combinations (Murugesan and Mahapatra 2022). The extender used in the study was a BPSE extender. Different extenders used, as well as cryoprotectant agents, can affect the study's results. DMEM extender was used in our study. DMEM extender is used in studies on songbird sperm (Humann‐Guilleminot et al. 2019; Poignet et al. 2022). Our study showed that EG was more successful on spermatological parameters than DMSO, which was used at the same rate. Bird sperm has a high density and a low seminal plasma ratio. Frozen sperm storage and thawing cause intracellular osmotic and thermal stress due to intracellular and extracellular ice crystals. Seminal plasma contains protein, carbohydrates, lipids, amino acids, various hormones and enzymes. It is thought that various components in seminal plasma may have a partial protective effect against these adverse effects (Santiago‐Moreno and Blesbois 2020). However, the low seminal plasma ratio in bird semen may be one of the reasons why spermatological parameters are lower than expected during cryopreservation and thawing of semen.

## 5 Conclusion

Unfortunately, the number of songbirds at risk of extinction is increasing. Canary semen was used because the canary is a domesticated songbird; it is easily available, and its sperm is similar to other songbirds. The effects of two different cryoprotectant agents were examined in the freezing of canary sperm. It was observed that 8% EG was more successful than 8% DMSO cryoprotectant agent in frozen and thawed canary sperm. Our study is promising for future studies in this field. It would be appropriate to investigate the effects of different doses of EG and to consider different cryopreservation methods in the freezing procedure.

## Author Contributions

A.O.Ö., B.E. and E.A. were involved in the systematic search to identify all relevant studies, assessed the eligibility of each selected study and helped draft the manuscript. A.O.Ö., B.E. and E.A. visualized the experiments. A.O.Ö. and B.E. analysed data and wrote the initial draft. A.O.Ö. participated in performing statistical analysis. All authors read and approved the final manuscript.

## Ethics Statement

The present study was approved by the Ondokuz Mayıs University Animal Ethics Committee (2023/59).

## Conflicts of Interest

The authors declare no conflicts of interest.

### Peer Review

The peer review history for this article is available at https://www.webofscience.com/api/gateway/wos/peer-review/10.1002/vms3.70101.

## Data Availability

The datasets used and analysed for this research are available from the corresponding author (A.O.Ö.) upon reasonable request.
